# Slaughterhouse Pigs Are a Major Reservoir of *Streptococcus suis* Serotype 2 Capable of Causing Human Infection in Southern Vietnam

**DOI:** 10.1371/journal.pone.0017943

**Published:** 2011-03-28

**Authors:** Ngo Thi Hoa, Tran Thi Bich Chieu, Tran Thi Thu Nga, Nguyen Van Dung, James Campbell, Pham Hong Anh, Huynh Huu Tho, Nguyen Van Vinh Chau, Juliet E. Bryant, Tran Tinh Hien, Jeremy Farrar, Constance Schultsz

**Affiliations:** 1 Oxford University Clinical Research Unit, Hospital for Tropical Diseases, Ho Chi Minh City, Vietnam; 2 Sub-Department of Animal Health, Ho Chi Minh City, Vietnam; 3 Hospital for Tropical Diseases, Ho Chi Minh City, Vietnam; 4 Centre for Tropical Medicine, Oxford University, Oxford, United Kingdom; 5 Academic Medical Centre, Centre for Poverty-related Communicable Diseases, University of Amsterdam, Amsterdam, The Netherlands; University of Edinburgh, United Kingdom

## Abstract

*Streptococcus suis* is a pathogen of major economic significance to the swine industry and is increasingly recognized as an emerging zoonotic agent in Asia. In Vietnam, *S. suis* is the leading cause of bacterial meningitis in adult humans. Zoonotic transmission is most frequently associated with serotype 2 strains and occupational exposure to pigs or consumption of infected pork. To gain insight into the role of pigs for human consumption as a reservoir for zoonotic infection in southern Vietnam, we determined the prevalence and diversity of *S. suis* carriage in healthy slaughterhouse pigs. Nasopharyngeal tonsils were sampled from pigs at slaughterhouses serving six provinces in southern Vietnam and Ho Chi Minh City area from September 2006 to November 2007. Samples were screened by bacterial culture. Isolates of *S. suis* were serotyped and characterized by multi locus sequence typing (MLST) and pulse field gel electrophoresis (PFGE). Antibiotic susceptibility profiles and associated genetic resistance determinants, and the presence of putative virulence factors were determined. 41% (222/542) of pigs carried *S. suis* of one or multiple serotypes. 8% (45/542) carried *S. suis* serotype 2 which was the most common serotype found (45/317 strains, 14%). 80% of serotype 2 strains belonged to the MLST clonal complex 1,which was previously associated with meningitis cases in Vietnam and outbreaks of severe disease in China in 1998 and 2005. These strains clustered with representative strains isolated from patients with meningitis in PFGE analysis, and showed similar antimicrobial resistance and virulence factor profiles. Slaughterhouse pigs are a major reservoir of *S. suis* serotype 2 capable of causing human infection in southern Vietnam. Strict hygiene at processing facilities, and health education programs addressing food safety and proper handling of pork should be encouraged.

## Introduction

Infections of *Streptococcus suis* constitute a major health problem in the swine industry worldwide. In neonatal and infant pigs, *S. suis* is the cause of outbreaks of septicaemia, meningitis, arthritis and pneumonia whilst in adult pigs asymptomatic nasopharyngeal carriage is well documented. *S. suis* is also an emerging zoonotic pathogen in Asia, and is increasingly recognized as a leading cause of bacterial meningitis cases and septicaemia in adult humans [1], [2], [3], [4]. Among the 33 described serotypes, serotype 2 is most frequently associated with invasive disease in both pigs and humans [5], [6]. Additional serotypes reported to cause invasive disease in pigs include serotypes 1 to 9, 1/2 and 14 [1], [3]. However, these strains are rarely found in human infection. Reported risk factors for *S. suis* infection in humans suggest the importance of both occupational and food-borne transmission [5], [7]. We identified consumption of pork with a high risk of contamination with *S. suis* as an independent risk factor for *S. suis* meningitis in southern Vietnam (Nghia *et al*, submitted for publication). Consumption of undercooked pork was identified as the most common risk factor in a retrospective cohort study in 66 patients infected with *S. suis* in Thailand [7].

Genetic characterization of *S. suis* serotype 2 strains isolated from human patients with meningitis in southern and central Vietnam, showed a highly conserved population structure [2]. In addition, these strains all carried the genes encoding the putative virulence factors Extracellular Protein Factor (EF) or one of its larger size variants EF*, and the hemolysin (suilysin), whilst 69.6% of these strains produced Muramidase Released Protein (MRP) [2], [8], [9], [10], [11]. These findings indicate the existence of a virulent clone of *S. suis* serotype 2, capable of persisting within the pig population and with a propensity to infect humans. Alternatively, the findings suggest a predominance of *S. suis* serotype 2, ST1 amongst the *S. suis* serotypes and sequence types prevalent in the southern Vietnamese pig population. To gain insight into the population structure of *S. suis* carried in healthy pigs, in particular pigs for human consumption which may constitute a reservoir for zoonotic infection, we determined the prevalence of *S. suis* serotype 2 and other serotypes in nasopharyngeal tonsils of slaughterhouse pigs in southern Vietnam. Strains were then subjected to molecular genetic characterization to further delineate population structure and to facilitate comparisons between the porcine isolates and representative human clinical isolates from southern and northern Vietnam.

## Materials and Methods

### Ethical Statement

The study protocol was reviewed and approved by the Sub Department of Animal Health of Ho Chi Minh City (1242/SNN-NN). All sampling was performed under the supervision of government staff following standard rendering procedures.

### Tonsil collection

**Figure 1:**
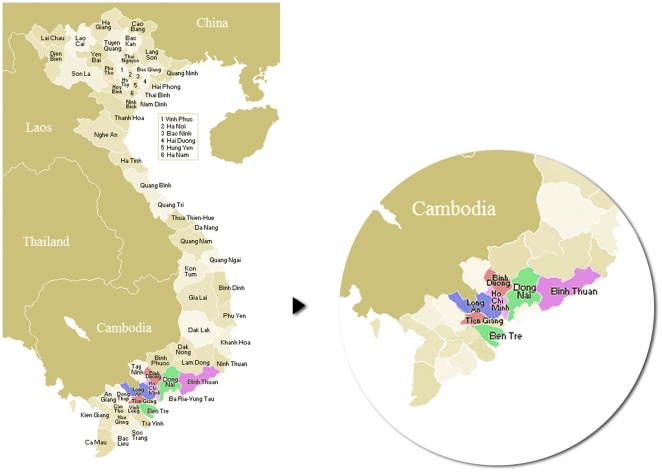
Provinces of Vietnam. Provinces (coloured) from which sampled pig originated are located in the south of Vietnam and surrounding Ho Chi Minh City.

Tonsils were collected prospectively from pigs submitted to three slaughterhouses receiving animals from 6 provinces in southern Vietnam (Dong Nai, Ben Tre, Binh Duong, Binh Thuan, Long An, Tien Giang) and from the greater Ho Chi Minh City area (Hoc Mon and Cu Chi) (Figure 1). The number of pigs slaughtered on a daily basis at each slaughterhouse varied between 40–50 and 800–1000.

Pigs were inspected for gross health status and were kept in groups according to delivery by the seller, for a maximum of 16 hours after arrival at the slaughterhouse. The age and province of origin of each pig was recorded. Visually healthy pigs were killed by electrocution in group based batches. Directly after decapitation, the pharyngeal tonsil was removed aseptically and transferred to the laboratory in separate sterile container on ice within 8 hours and stored at −20°C until culture.

Each slaughterhouse was sampled once during repetitive sampling rounds of four-weeks. To ensure sampling from multiple herds originating from multiple provinces, a maximum of 50 tonsils from consecutively slaughtered pigs was collected during each sampling occasion at each slaughterhouse, until a minimum total of 500 tonsils was collected. Sample size calculations indicated that a sample size of 500 tonsils is sufficient to reliably estimate *S. suis* carriage rates. An observed carriage rate of 50% would result in an exact 95% confidence interval (CI) of 45.5 to 54.4%, whilst a rate of 5% would result in a 95% CI of 3.3 to 7.3%.

### Tonsil culture

The inner section of each tonsil was minced on a sterile petri dish. A loop of minced tonsil was streaked onto blood agar plates containing Streptococcal Selective Reagent (Oxoid, UK) and crystal violet. Plates were incubated at 37°C in 5% CO_2_ for 16–18 hours. A maximum of six colonies suspected for *S. suis* was selected from each plate. *S. suis* was identified on the basis of colony morphology and alpha-hemolysis, Gram-stain, catalase reactivity, and biochemical identification using API20Strep (BioMerieux, Vietnam). Serotyping was performed by slide agglutination using antisera against all 33 polysaccharide capsule antigens (Statens Serum Institute, Denmark). Multiple colonies of different morphology were tested from each tonsil sample. Isolates were screened using polyvalent antisera, followed by agglutination with appropriate monovalent antisera. Strains *S. suis* 31533 and *Lactococcus lactis spp lactis* strain FX256-6 were used for quality control purpose.

### Molecular typing

All non-duplicate *S. suis* serotype 2 strains were characterized by multi locus sequence typing (MLST) and pulse field gel electrophoresis (PFGE) as described previously [2], [12]. The multi locus sequence type assignment was based on the sequence of the alleles at each locus of 7 housekeeping genes included in the MLST scheme, using the MLST database [13]. Primer sequences used were as described and published on the MLST website (www.mlst.net), except for the *recA* and *mutS* alleles for which adjusted primers were designed with the following sequences; ss*recA*-f: ATAGCCACGTTGGTTTGCAG, ss*recA*-r: CATAATGAACACGTATCTTGCGG; ss*mutS*-f GAGCAGATGGAAGATCCTAAGC, ss*mutS*-r ACAAACTACCATGCTTCTTGCC. MLST results were analyzed using eBURST (www.mlst.net) [13].

PFGE with *Sma*I digestion was performed as described elsewhere [2] and the images of gels were analysed using Bionumerics (Applied Maths, Belgium). Six strains, each representing one of the six predominant PFGE types which were identified in *S. suis* serotype 2 strains isolated from human patients with meningitis in southern and northern Vietnam [2], [14], were included in the PFGE analysis to facilitate comparison of strains isolated from humans and pigs.

### Virulence factor profiling

The presence of genes encoding the putative virulence associated factors EF (epf/epf*), and suilysin (*sly*) was determined for all strains belonging to the predominant serotypes found, using multiplex PCR as described [15]. MRP expression was determined by Western blot for all serotype 2 strains using polyclonal antibodies against MRP (provided by H. Smith) [16]. *S. suis* serotype 2 strains 31533 and 89–1591 were used as positive and negative controls, respectively.

### Antimicrobial resistance profiling

Antimicrobial susceptibility testing was done on all *S. suis* serotype 2 isolates using E-test (AB Biodisk, Sweden), according to instruction of the manufacturer. The antimicrobials tested were penicillin, chloramphenicol, erythromycin, tetracycline, ciprofloxacin and vancomycin. Interpretative criteria were used according to CLSI guidelines for viridans group streptococci [17]. *Streptococcus pneumoniae* strain ATCC 49619 was used for quality control purpose. Erythromycin resistance phenotypes were identified using the triple disk diffusion test as described [18].

Strains resistant to tetracycline and/or erythromycin were further characterized by detection of the *erm*(A), *erm*(B), *mef*(A), *tet*(M), *tet*(O), *tet*(L) *and tet*(K) genes using multiplex PCRs as described by Malhotra–Kumar *et al* [19]. Strain BM407 containing *tet*(M), *tet*(O), *tet*(L) and *erm*(B) was used as a positive control [20]. Primers Tet(W)-F-HN (GGTGCAGTTGGAGGTTGTTT); Tet(W)-R-HN (CCTTCAATGCCTGTTCCAAT) and tet(W)- F (TTGGAATTCTTGCCCATGTAGACGC); tet(W)- R (TTGTCCAGGCGGTTGTTTGGAC) were designed to detect the fragment of *tet*(W) and the full length gene, respectively. The presence of the gene was confirmed by sequencing of the full length amplicons. The presence of the mosaic gene *tet*(O/W/32/O) was searched for in strains which produced PCRs amplicons for detection of fragment of *tet*(W) and the full length *tet*(O), using primers tet(O)-F-pDG364 (ATGAAAATAATTAACTTAGG) and tet(O)-R-pDG364 (TTAAGCTAACTTGTGGAACA *)*. The DNA sequence of the latter PCR amplicons was determined to confirm the presence of *tet*(O/W/32/O).

## Results

### Tonsil culture

Nasopharyngeal tonsils from 542 pigs were collected between September 2006 and November 2007 from six southern provinces and the Ho Chi Minh City metropolitan area (Table 1). Three hundred and four (56%) pigs originated from large-scale production units with herd sizes more than 500 head; 142 (26%) pigs originated from smaller semi-intensive farms with average herd sizes of 200 to 500 head, and 96 (18%) pigs were from backyard farms with less than 200 head. The majority of pigs (513, 94.6%) were between 3.5 and 6 months old, and the remaining (29, 5.4%) were sows.

**Table 1:** Sampling of tonsils and associated culture results.

Slaughter-house	Number of sampling round	Number of provinces samples *	Total number of tonsils collected	Number of tonsils with *S. suis* n (%)	Number of tonsils with S. suis serotype 2n (%)	Number of S. suis isolates
I	3	3	131	59 (45)	10 (16.9)	84
II	5	5	240	95 (39.6)	25 (26.3)	147
III	5	3	171	68 (39.8)	10 (14.7)	86
Total	13	7	542	222 (40.9)	45 (20.3)	317

*Pigs sampled in slaughterhouse I originated from (number of samples): Ben Tre province (25), Binh Duong province (31), Dong Nai province (75); slaughterhouse II: Binh Duong province (55), Binh Thuan province (83), Ho Chi Minh City (Hoc Mon) (12), Long An province (30), Tien Giang province (34); slaughterhouse III: Binh Duong province (50), Ho Chi Minh City (Cu Chi) (84), Long An province (37).

Cultures of 73 tonsil samples could not be interpreted due to overgrowth of bacteria other than *S. suis*. *S. suis* was recovered from 222 (41%) of 542 tonsils sampled. These 222 tonsils yielded a total of 317 individual *S. suis* strains (Table 1). Amongst these 317 strains, serotype 2 was the most common (45 strains, 14.2%), followed by strains that (repeatedly) reacted with all three antisera against serotypes 9, 31 and 32 (38 strains, 12%) (Table 2). Other predominant serotypes identified included serotypes 3, 21, 16 and 4. Forty-five strains (14.2%) were untypeable (Table 2).

**Table 2:** Serotype distribution across *Streptococcus suis* strains isolated from pig tonsils.

	Serotype distribution (N = 317)	
Serotype	No. of strains	%
2	45	14.2
9,31,32@	38	12
3	27	8.5
21	20	6.3
7	17	5.4
16	12	3.8
4	10	3.2
Other*	65	20.5
Other, double serotypes**	29	9.2
Other, triple serotypes**	9	2.8
Untypeable#	45	14.2

@ Serotyping was repeated three times; additional analysis of 7 representative strains by MLST and 16S rDNA sequencing confirmed that these strains are S. suis.

*Includes serotype 5, 8, 9,10, 11, 12, 13, 15, 19, 20, 22, 23, 25, 26, 27, 28, 29, 30, 31, 33.

**Strains reacting with antisera against two or three different serotypes.

#23 strains were auto-agglutinating, 16 strains could not be assigned to any serotype after agglutination with polyvalent antisera, and 6 strains reacted negatively with all poly-valent antisera.

*S. suis* carrier pigs were identified from all provinces studied (Figure 1). Prevalence rates of *S. suis* carriage of all serotypes and of *S. suis* serotype 2 specifically varied from 14.7% to 48.5% and 5% to 20% respectively, across provinces. Nineteen (6.3%) *S. suis* serotype 2 strains were isolated from 304 pigs reared in intensive farms;19 (13.4%) from 142 pigs reared in semi-intensive farms, and 7 (7.3%) strains were isolated from 96 pigs reared in backyard farms. The proportion of *S. suis* serotype 2 strains originating from semi-intensive farms was significantly higher than those from either intensive or small farms (p<0.021, Pearson's chi-square test).

### Characterization of *S. suis* serotype 2 strains

MLST analysis indicated that 36 of 45 (80%) *S. suis* serotype 2 strains were sequence type 1 (ST1). On eBURST analysis ST1 was the most commonly found sequence type, representing clonal complex 1 (CC1). One single locus variant of ST1 was identified based on detection of a new allele for *recA*, and this strain was assigned to ST158. Nine strains (20%) were assigned to ST28, belonging to CC27.

**Figure 2:**
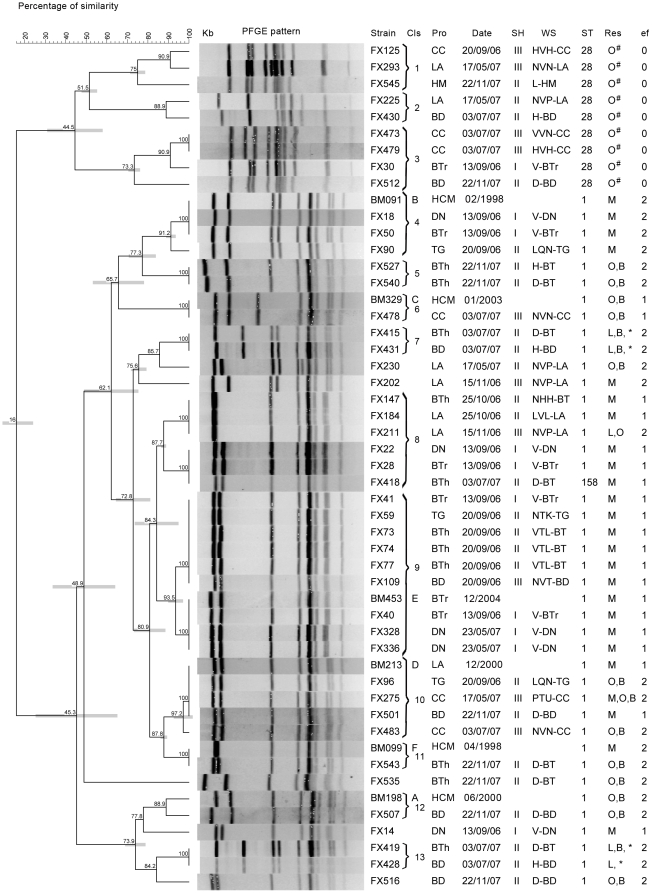
Pulse field gel electrophoresis (PFGE) of *Streptococcus suis* serotype 2 strains isolated from tonsils of healthy pigs. Six *S. suis* serotype 2 strains isolated from patients with meningitis in southern Vietnam whose PFGE patterns represent the dominant PFGE patterns across *S. suis* serotype 2 strains isolated from humans (labeled A–F), were included for comparison purpose. A dendrogram was generated by Dice analysis (band tolerance, 1.3%) and cluster analysis with unweighted pair group method with arithmetic mean, using Bionumerics software (Applied Maths, Belgium). Bars indicate 95% confidence intervals. Columns: Cls: PFGE cluster; Pro: province of pig's origin or patients' residence; Date: date of tonsil collection or patients' admission; SH: slaughterhouse ID; WS: Whole sale seller ID; ST: sequence type, Res: tetracycline and erythromycin resistance gene detected by PCR and sequencing, ef: *epf* or epf* gene detected by PCR. CC: Cu Chi- HCMC; LA: Long An; HM: Hoc Mon – HCMC; HCM: Ho Chi Minh City; BD: Binh Duong; BTr: Ben Tre; DN: Dong Nai; BTh: Binh Thuan. O: amplicons of *tet*(O) gene detected. M: amplicons of *tet*(M) gene detected. B: amplicons of *erm*(B) gene detected. L: amplicons of *tet*(L) gene detected *: amplicons of mosaic tetracyclin resistance encoding gene *tet*(O/W/32/O) detected in these strains. #: no amplicons of *erm*(A), *erm*(B) or *mef*(A) were detected in these erythormycine resistant strains. 0: no amplicons of *epf* or epf* gene detected. 1: amplicons of *epf* gene detected. 2: amplicons of epf* gene detected.

On PFGE analysis, strains could be assigned to two major clusters, corresponding with the ST distribution. To facilitate comparison with strains isolated from human patients, 6 *S. suis* serotype 2 strains of ST1 isolated from human patients in southern Vietnam were included in the PFGE analysis. These strains (indicated by A–F in Figure 2) represent the 6 major PFGE clusters observed during previous PFGE analyses of 128 strains of *S. suis* serotype 2 isolated from human patients in southern and northern Vietnam [2], [14]. All human strains clustered together with isolates from pig tonsils. Four additional clusters (clusters 5, 7, 8 and 13 in Figure 2) contained only strains isolated from pig tonsils. Identical band patterns were observed for strains isolated from pigs reared in different provinces (cluster 4), for strains isolated from pigs slaughtered in different slaughterhouses (cluster 9), and for strains isolated from pigs sampled in different sampling rounds (cluster 10), indicating the clonal spread of epidemiologically unrelated *S. suis* serotype 2 strains of ST1. However, differences in resistance determinant profiles were observed for strains with identical band patterns within PFGE clusters 8, 10, 11 and 13 (Figure 2). Among the 9 strains of ST28, 3 clusters were observed in PFGE analysis (Figure 2). Similar to the strains of ST1, pigs which carried these 9 strains originated from different provinces and were sampled in different sampling rounds.

All 45 *S. suis* serotype 2 strains were fully susceptible to penicillin, vancomycin and ciprofloxacin but were resistant to tetracycline. Resistance to erythromycin was observed in 23 (51%) strains. Resistance to chloramphenicol, including intermediate resistance, was found in 12 (26.7%) strains. The tetracycline resistance determinants *tet(*M), *tet*(O) and *tet*(L) were amplified in 21 (46.7%), 21 (46.7%), and 5 (11.1%) strains, respectively. Single tetracycline resistance encoding genes (*tet*(M) or *tet*(O)) were detected in 39 (86.7%) strains. Four strains contained full-length *tet*(O/W/32/O) genes, with sequences 100% identical to those described for these genes in *S. suis* strains isolated from pigs in Italy (NCBI accession number FM164392) [21]. Combinations of tetracycline resistance encoding genes, including *tet*(O) and *tet*(L), *tet*(O/W/32/O) and *tet*(L), or *tet*(M) and *tet*(O), were found in 6 (13.3%) strains. Amplicons of the genes *tet*(K) and *tet*(W) were not found. Twenty (87%) strains expressed the erythromycin resistance cMLS_B_ phenotype and three (13%) strains expressed the M phenotype. The gene *erm*(B) was found in 14 (60.9%) erythromycin resistant strains. Four different combinations of the *erm*(B) gene with tetracycline resistance genes were found, including *erm*(B) and *tet*(O) (10 strains); *erm*(B), *tet*(O) and *tet*(M) (1 strain); *erm*(B), *tet*(L) and *tet*(O/W/32/O) (3 strains). We were unable to amplify amplicons of *erm*(A), *erm*(B) or *mef*(A) genes in 9 (39.1%) erythromycin resistant strains and in the remaining 22 erythromycin susceptible strains.

All 36 *S. suis* serotype 2 strains of ST1 possessed the *epf+/sly+* or epf*+/sly+ genotype. In contrast, none of the 9 *S. suis* serotype 2 strains of ST28 possessed this genotype. The expression of muramidase released protein (MRP) was observed in 34 of 45 *S. suis* serotype 2 strains, including all 9 strains of ST28. For comparison, the prevalence of these virulence associated genes was also determined in all strains of the predominant other serotypes found. Only 4 of 125 (3.2%) strains with serotypes (9,31,32), 3, 21, 7, 16, 4 or 9 possessed the *epf+/sly+* or epf*+/sly+ genotype. Amplicons of either the *epf*, epf* or *sly* genes were detected in 28 (22.4%) of these strains.

## Discussion

We studied the prevalence of *S. suis* in slaughterhouse pigs in southern Vietnam by systematic sampling of pig tonsils in three slaughterhouses which received pigs for slaughter from different farms in 6 provinces and HCMC area in southern Vietnam. We were particularly interested in the prevalence of *S. suis* serotype 2 strains compared to other serotypes as strains carrying the serotype 2 polysaccharide capsule are responsible for the vast majority of human infections in Vietnam as well as worldwide [1], [6].

We observed an overall *S. suis* carriage rate of 41% among pigs entering slaughter facilities in southern Vietnam. The prevalence rate of carriage of the pathogenic serotype 2 was 8%. Colonization of pigs with *S. suis* occurs at an early stage of life, often through vertical transmission from carrying sows. *S. suis* carriage rates may vary between herds and can range from 0% to up to 80-100% [22], [23], [24] The finishing pigs sampled in our study were aged between 3.5 to 6 months and originated from multiple farms in multiple provinces in southern Vietnam. Therefore, the carriage rates of 41% for *S. suis* and 8% for *S. suis* serotype 2, are likely to represent a reliable estimate of *S. suis* carriage prevalence in pigs raised in the region during the sampling period. Our prevalence estimates were based on bacterial culture rather than molecular screening methods, and are thus likely to be a conservative estimate of true prevalence [25]

*S. suis* serotype 2 was the most common serotype isolated from the sampled pigs, indicating that *S. suis* serotype 2 is highly prevalent in slaughterhouse pigs in southern Vietnam. In contrast, in a study of slaughterhouse pigs in Korea, *S. suis* serotype 2 strains were absent, whilst serotype 9 was the most common serotype [26]. A large survey amongst 1043 healthy sows in 10 regions in China showed a similar *S. suis* carriage rate (40.4%) but a lower carriage rate of serotype 2 strains (3.0%) [27]. *S. suis* serotype 2 strains of MLST CC1 are considered to represent a population of particularly virulent strains causing invasive infection in both pigs and humans [1], [2], [12]. All *S. suis* serotype 2 strains isolated from humans in southern Vietnam for which MLST was reported, belonged to this clonal complex, as well as the strains causing outbreaks in China in 1997 and 2005 [2], [4], [28]. All *S. suis* serotype 2 strains of ST1 in this study were positive in a PCR detecting the presence of the virulence-associated genes epf/epf* and *sly* and almost 70% of these strains expressed MRP. These results are consistent with our observations in strains isolated from adult human patients [2]. In addition, strains representing the prevalent genotypes of *S. suis* serotype 2 isolated from humans, all clustered with the pig carriage isolates on PFGE analysis.

Despite the clonal structure of the *S. suis* serotype 2 population under study, differences were observed in the antimicrobial resistance determinants content of strains, in particular for genes encoding tetracycline and erythromycin resistance. We observed multiple combinations of tetracycline resistance encoding genes, including the presence of *tet*(L), in the presence or absence of genes encoding erythromycin resistance (Figure 2). These results indicate circulation of multiple descendants of MLST ST1 lineage in multiple provinces in southern Vietnam.

Human infections with *S. suis* serotype 2 strains of MLST CC27, in particular strains with ST28, have been reported from Japan and Thailand but to date have not been reported from Vietnam or China [29], [30]. The ST28 strains present in our study did not possess the virulence-associated marker genes *epf* and *sly*, suggesting that reduced virulence may explain their absence amongst strains causing human infections in Vietnam. However, the possible emergence of ST28 strains in human clinical cases in Vietnam cannot be ruled out considering their presence within the slaughterhouse pig population.

We observed a high prevalence of *S. suis* strains which reacted with antibodies against capsular antigens of serotypes 9/31/32. Strains were identified using APIStrep, a well validated phenotypic identification method. However, as it is known that *S. suis* carriage strains are diverse and may be untypeable, additional genotypic confirmation methods for identification are recommended [1]. We therefore studied 7 randomly selected strains in more detail. MLST and16S rDNA sequencing results further confirmed that these strains are indeed *S. suis* (data not shown). MLST results also suggested that the population of strains reacting with all three of the 9, 31, and 32 antibodies is heterogeneous and it is likely that these strains represent a diverse population carried in healthy pigs (data not shown). None of the strains possessed any of the genes typically used as virulence markers of *S. suis*. Despite their relatively high prevalence in slaughterhouse pigs in southern Vietnam, strains of serotype 9, 31, 32 have never been reported from human patients in the region nor in human patients in other geographical areas, supporting their presumed limited virulence.

Other serotypes found amongst the *S. suis* carriage isolates included serotype 3, serotype 21, serotype 7, serotype 16 and serotype 4. Of these, only serotype 16 has been reported in a human patient [31]. Serotype 3 strains were found amongst *S. suis* strains causing invasive infection in pigs in China [32] but have never been reported to cause disease in humans. Despite its occurrence in human infection in southern Vietnam, as well as in Thailand and North America [33], [34], [35], strains of serotype 14 were not found in our study population. To the best of our knowledge, this is the first study reporting the prevalence of *S. suis* in healthy pigs in Vietnam and data on the most prevalent serotypes causing invasive disease in Vietnam are lacking.

In conclusion, our study shows that *S. suis* carriage is common in healthy slaughterhouse pigs in southern Vietnam and that serotype 2 is the most prevalent serotype. *S. suis* serotype 2 strains found in slaughterhouse pigs are highly similar to those isolated from human patients indicating that pigs for consumption could be a source of human infection. Human exposure to virulent *S. suis* strains through handling of pork for consumption may be particularly hazardous in an environment where food items are kept at high ambient temperatures, resulting in high bacterial counts, as is the case in southern Vietnam. Therefore, health education programs addressing the proper handling of pork and other pig products should be encouraged.
